# Mitochondrial turnover in liver is fast *in vivo* and is accelerated by dietary restriction: application of a simple dynamic model

**DOI:** 10.1111/j.1474-9726.2008.00426.x

**Published:** 2008-12

**Authors:** Satomi Miwa, Conor Lawless, Thomas von Zglinicki

**Affiliations:** Centre for Integrated Systems Biology of Ageing and Nutrition, Institute for Ageing and Health, Newcastle UniversityNewcastle upon Tyne, UK

**Keywords:** dietary restriction, half life, liver, mathematical model, mitochondria, mice, turnover

## Abstract

‘Mitochondrial dysfunction’, which may result from an accumulation of damaged mitochondria in cells due to a slowed-down rate of mitochondrial turnover and inadequate removal of damaged mitochondria during aging, has been implicated as both cause and consequence of the aging process and a number of age-related pathologies. Despite growing interest in mitochondrial function during aging, published data on mitochondrial turnover are scarce, and differ from each other by up to one order of magnitude. Here we demonstrate that re-utilization of the radioactively labelled precursor in pulse-chase assays is the most likely cause of significant overestimation of mitochondrial turnover rates. We performed a classic radioactive label pulse-chase experiment using ^14^C NaHCO_3_, whose ^14^C is incorporated into various amino acids, to measure mitochondrial turnover in mouse liver. In this system, the activity of the urea cycle greatly limited arginine dependent label re-utilization, but not that of other amino acids. We used information from tissues that do not have an active urea cycle (brain and muscle) to estimate the extent of label re-utilization with a dynamic mathematical model. We estimated the actual liver mitochondrial half life as only 1.83 days, and this decreased to 1.16 days following 3 months of dietary restriction, supporting the hypothesis that this intervention might promote mitochondrial turnover as a part of its beneficial effects.

Recent years have seen a surge of interest in the role of mitochondrial dysfunction, reactive oxygen species production and mitochondrial DNA mutation as driving factors in the aging process (Balaban *et al*., 2005; Trifunovic *et al*., 2005; Bender *et al*., 2006; Passos *et al*., 2007). Damaged mitochondria might accumulate in cells due to a slowed-down turnover with aging (de Grey, 1997; Kowald & Kirkwood, 2000; Terman & Brunk, 2005). Conversely, it was proposed that the ‘anti-aging’ function of dietary restriction (DR) might be at least partially due to stimulation of molecular and, specifically, mitochondrial turnover (Donati *et al*., 2001; Bergamini *et al*., 2003; Del Roso *et al*., 2003; Cuervo *et al*., 2005). Surprisingly, essentially all information on mitochondrial turnover relies on data that are not only at least 20 years old but that vary by one order of magnitude between different estimates. These differences are largely methodological. Mitochondrial protein degradation is generally measured by radio-isotope pulse-chase assay to calculate the rate constant of degradation (and so the half life), assuming that the data follow a simple exponential decay. Label re-utilization (i.e. the re-incorporation of labelled precursor arising from broken down products of the pulse-labelled protein) can be a major problem with this approach, leading to significant lengthening of the apparent half life. An ideal precursor would allow fast protein labelling (i.e. the level of the specific activity of the precursor pool should quickly decrease to zero) thereby avoiding label re-utilization. In essence, the best precursor will give the shortest half life estimate.

Labelling of liver mitochondria with ^14^C NaHCO_3_ (which is converted to arginine in the liver) (Lipsky & Pedersen, 1981; Lavie *et al*., 1982; Saikumar & Kurup, 1985) or ^14^C arginine (Aschenbrenner *et al*., 1970; Gross, 1971; Glass & Doyle, 1972) has consistently given the shortest half life in contrast to the use of other amino acid precursors, suggesting that label re-utilization of non-arginine amino acid precursors was a major problem. This is because ^14^C NaHCO_3_ is converted to ^14^C arginine (the ^14^C label being in the guanidine position; 6-^14^C arginine) in the liver through urea cycle activity, while NaHCO_3_ turnover is fast *in vivo* and the specific activity of ^14^C NaHCO_3_ decreases rapidly (Millward, 1970). Re-utilization of labelled arginine is minimized by the high activity of arginase found in the liver, which quickly decomposes arginine into urea (which inherits the labelled ^14^C) that is readily excreted. Unlike ^14^C arginine, ^14^C NaHCO_3_ will not substantially label proteins in nonhepatic tissues and can thus avoid ^14^C label re-utilization of broken down products deriving from nonhepatic tissues.

However, in a standard ^14^C NaHCO_3_ pulse-chase protocol as described by Lipsky & Pedersen (1981, 1982) (Fig. 1A), we still found evidence for label re-utilization: the fit of a single exponential decay curve to the data was not good. In particular, using this model, the estimated half lives increased systematically if later time points were included in the data set (Fig. 1B). In addition, when comparing DR animals to controls, the estimated half lives appeared faster or slower depending on the length of the chase period (Fig. 1B). This indicated that the decay in specific activity of ^14^C in liver mitochondria followed a more complex pattern consisting of a fast component, which is the degradation of mitochondrial 6-^14^C-labelled arginine, and another slower component. The slow component could be non-arginine-derived-^14^C, which would not escape the re-utilization problem. In fact, a small fraction of exogenous carbon is incorporated into non-arginine amino acids in the liver, and this is similar to the incorporation in skeletal muscles and intestine (Swick *et al*., 1953; Swick & Handa, 1956), which do not have complete and active urea cycle enzymes to convert ^14^C NaHCO_3_ to 6-^14^C arginine. Accordingly, we found low ^14^C labels in skeletal muscles and brain mitochondria, which were similar to each other (Fig. 2A). Moreover, both the absolute values and the rates of label decay in muscle and brain mitochondria were very similar to the estimated slow component in liver mitochondria (estimated by a two-component decay model). Therefore, we propose to use data on the slow component obtained from different tissues of the same animals as a surrogate estimate for the slow component in the liver. This significantly improves the statistical power of the obtainable fit in comparison to a two-component decay model which is fit to liver data alone.

**Fig. 1:**
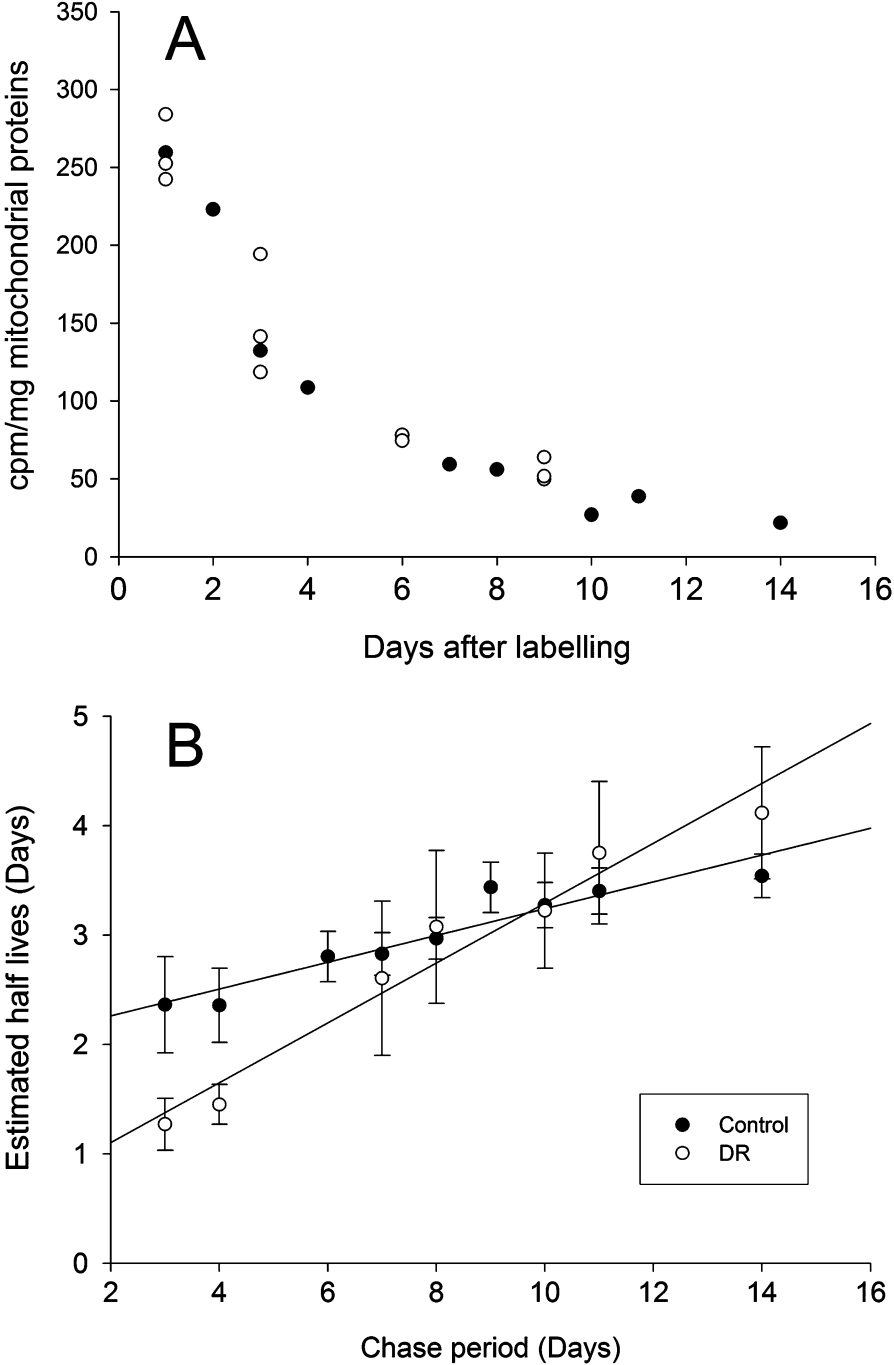
A single exponential decay model is not adequate to measure mouse liver mitochondrial half life. (A) Changes in specific activity of ^14^C in liver mitochondria from control mice with time. Results from two sets of independent experiments are shown (full and open circles). Each data point represents an individual animal. (B) Effect of chase period on half lives of mitochondria from dietary restricted (DR) and control mice. Apparent half life time of mitochondria λ depends on the chase period if calculated as single exponential decay by logarithmic transfer. Data are mean ± SEM. • = DR; 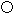 = controls. These estimates in control animals are in good agreement with published data with corresponding chase periods (Lipsky & Pedersen, 1981; Lavie *et al*., 1982; Saikumar & Kurup, 1985).

**Fig. 2:**
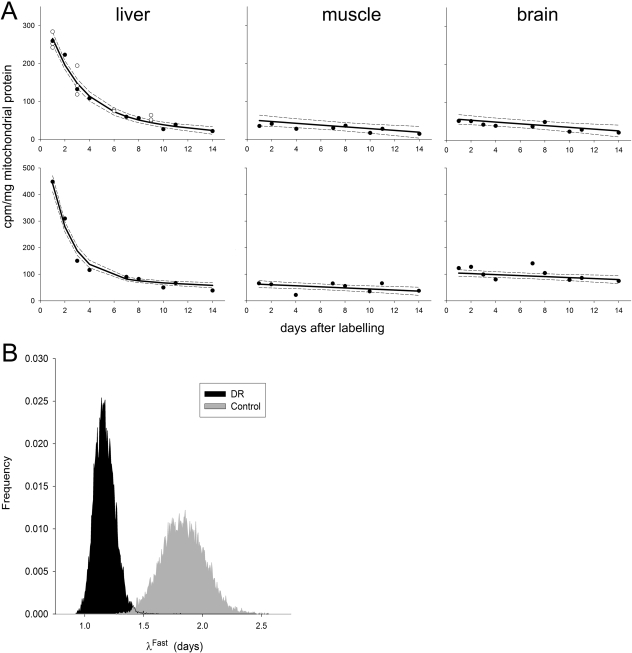
Liver mitochondrial half life is short and decreases further by dietary restriction. (A) Observed (circles) and median simulated (solid lines) decay of specific activity of ^14^C label in liver (left), muscle (centre) and brain (right) mitochondria. Top panels are from 6-month-old control mice and the bottom panels are from mice dietary restricted for 3 months. Dashed lines are 5% and 95% quantiles of samples from simulated label count posterior distributions (20 000 simulations sampled after discarding 10 000 ‘run-in’ simulations). (B) Posterior frequency distributions of calculated half lives (λ^Fast^, in days) of liver mitochondria from control and dietary restricted mice. In 99.96% of the 20 000 sampled simulations λ_*DR*_ *<* λ_*Control*_, which is a statistically significant result. The median values for λ_*DR*_ and λ_*Control*_ are 1.16 days and 1.83 days, respectively.

Thus, we describe nonspecific ^14^C label and its decay in liver mitochondria (the slow component) by the average of the brain and muscle ^14^C counts. Modelling the slow decay in all three tissues as a linear decrease, i.e. the simplest dynamic model possible (an exponential decay model changed the results very little), we obtain:


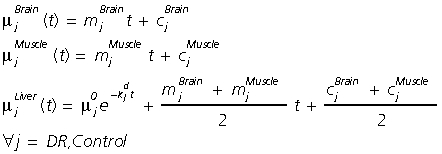


where 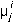 (count · mg^−1^) are the total ^14^C counts at time *t* (day) for tissue *i* and experimental condition *j* (control and DR), 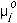 (count · mg^−1^) represents the value of the exponential ‘fast’ component in the liver at *t*= 0, 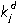 (day^−1^) are rate parameters describing the exponential decay of the specific label (6-^14^C arginine) with time in the liver, and 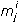 (count mg^−1^ · *t*^−1^) and 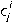 (count · mg^−1^) are the slope and intercept of the slower linear processes observed in tissue *i* and experimental condition *j*, respectively. Note that the amount of label in the liver at time *t*= 0 (the amount incorporated initially) can be estimated as:


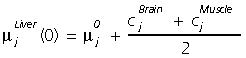


We estimated model parameter values for both control and DR mice by Bayesian inference using Gibbs sampling (using Markov chain Monte Carlo methods) as implemented in the OpenBUGS software package (Thomas *et al*., 2006) with starting conditions as indicated in the Supporting Information (script available: http://www.cisban.ac.uk/downloads/Miwa2008.odc). The main advantage of this method over more traditional ones such as least squares is that it generates parameter and model estimate distributions instead of just a single ‘best’ value. This allows us to test for the significance of differences observed between treatments (i.e. DR and control). Modelling process dynamics in all three tissues simultaneously allows us to use information in the brain and muscle data sets to increase the precision of the parameter estimates in the liver model beyond that which we would have using the liver data alone, and to assess the difference in liver mitochondrial half lives between control and DR mice more rigorously.

The simulations fitted the observed data in brain, skeletal muscle and liver mitochondria from both control and DR mice very well (Fig. 2a). Posterior half life distributions for the exponential-decay-component (the fast component, representing the decay of 6-^14^C arginine-dependent labels) in liver are shown in Fig. 2b. Thus, the estimated median half life of liver mitochondria is 1.83 days for controls and 1.16 days following 3 months DR, a statistically highly significant difference. Table S1 (Supporting Information) summarizes the parameter estimates and their distribution statistics.

Macro-autophagy is the major pathway of mitochondrial degradation (Kim *et al*., 2007), which is known to be accelerated by starvation. It has been proposed that DR might act similarly and, by promoting mitochondrial turnover, maintain a healthy population of mitochondria (Bergamini *et al*., 2003; Kim *et al*., 2007). We demonstrate here for the first time that DR animals indeed have significantly faster rate of liver mitochondrial turnover compared with controls. It should be noted, however, that starvation-induced autophagy is tissue specific. For example, fasting promoted autophagy in liver and skeletal muscles but not in brain (Mizushima *et al*., 2004). Thus, it is possible that increased turnover of mitochondria may be one of the beneficial mechanisms of DR in liver, but may not necessarily be so in all cell types.

It should also be noted that the concept of mitochondrial half life in itself is problematic. First, mitochondria form dynamic syncytia in cells. Second, macro-autophagy might be selective (for instance, for damaged mitochondria with low membrane potential). Third, at least some mitochondrial protein turnover is mediated by mitochondrial matrix (Lon) proteases (Bulteau *et al*., 2006; Ngo & Davies, 2007). Our estimates constitute averages over all the heterogeneity resulting from these various processes.

## Supporting information

**Appendix S1** Supplementary experimental procedures

**Table S1** Parameter estimates and their distribution statistics.
