# Supplementary material for: Mitochondrial turnover in liver is fast in vivo and is accelerated by dietary restriction: application of a simple dynamic model
Source: Aging Cell. 2008 Dec;7(6):920–3. doi: 10.1111/j.1474-9726.2008.00426.x (PMC2659384; doi:10.1111/j.1474-9726.2008.00426.x)
Supplement: Supplementary file 1 [file ace0007-0920-SD1.doc]

**Supplementary Experimental procedures**

1. **Chemicals**

^14^C sodium bicarbonate (^14^C NaHCO_3_) was purchased from GE Healthcare, UK. Other chemicals were from Sigma.

1. **Animals**

C57Bl6 male mice were used in the experiments and fed a CRM (p) diet (Special Diets Services, Essex, UK).

Dietary restriction (DR) of 40% restriction compared with ad libitum controls was started at the age of 3 months and continued for 11 weeks. All the mice had free access to water, and were caged in a group of 3 to 5 animals.

1. **Radioisotope pulse chase protocol**

^14^C NaHCO_3_ was diluted in 0.9% saline solution, and each mouse received 300 μl of the ^14^C NaHCO_3_ -saline solution containing the specific activity 54 μCi by intraperitoneal injection. ^14^C labelled bicarbonate is converted into arginine (at the guanidine position) in liver by the urea cycle enzymes. The liver has high arginase activity decomposing arginine into urea and ornitine (Swick & Song, 1974). Urea is formed from the guanidine position of arginine, thus, the radiolabel is efficiently removed from the system (Swick, 1958). ^14^C NaHCO_3_ as label in liver experiments is advantageous over direct administration of ^14^C arginine because 1) non-hepatic tissues will not be substantially labelled (McFarlane, 1963) thus reducing ^14^C label re-utilization due to broken down products originating from non-hepatic tissues (for example, compared to ^14^C arginine, use of ^14^C NaHCO_3_ resulted in 30% shorter estimated half life of plasma albumin, a product of liver (Swick & Ip, 1974)) and 2) its turnover is rapid (Millward, 1970).

After the injection the mice were swiftly returned back to negative ventilation cages and kept as normal (for the DR mice, the same feeding was continued until the end of the study). It is assumed that mitochondrial turnover was in a steady state throughout the experimental period, and there was no significant variation in the mitochondrial yield within a group. The mice were sacrificed by cervical dislocation at the indicated time points. The tissues were taken immediately, weighed, and mitochondria were isolated. The radioactivity remaining in isolated mitochondria was counted by a liquid scintillation counter. The mitochondrial protein concentration was determined using BioRad *Dc* protein concentration assay kit, using BSA as a standard.

1. **Isolation of mitochondria**

Liver mitochondria were isolated as described (Chappell & Hansford, 1972) in medium comprising 0.25 M sucrose, 5 mM Tris/HCl and 2 mM EGTA (pH 7.4 at 4 °C). The quality of isolated liver mitochondria was controlled by measuring the respiratory control ratio (3.3) and the rate of oxygen consumption (86 nmol O min^-1^.mg protein^-1^ (state 3) and 26 nmol O min^-1^.mg protein^-1^ (state 4) with succinate as a substrate).

Skeletal muscle mitochondria were isolated as previously described (Cadena*s et* al., 2002) with minor modifications. The skeletal muscle was dissected from the hindlimbs, shredded with a sharp blade, minced with sharp scissors, and rinsed with isolation medium containing 100 mM KCl, 50 mM Tris-HCl, 2 mM EGTA (pH 7.4 at 4 °C)., The tissue was transferred to a medium containing 100 mM KCl, 50 mM Tris-HCl, 2 mM EGTA, 1 mM ATP, 5 mM MgCl_2_, 0.5% (w/v) bovine serum albumin (BSA), and 18.7 units of protease/g of tissue (Type VIII Subtilisin A -Sigma P5380) (pH 7.4 at 4 °C) and stirred for 3 min, then homogenized with a glass tissue homogenizer. The homogenate was centrifuged at 490 × g for 10 min. The supernatant was filtered through muslin and centrifuged at 10,368 × *g* for 10 min. Mitochondrial pellets were resuspended in isolation medium, and centrifuged at 10,368 × *g* for 10 min and then at 3841 × *g* for 10 min, and resuspended in isolation medium.

Brain mitochondria were isolated according to (Lai & Clark, 1979). All mitochondrial isolation procedures were carried out at 4 °C.

1. **A simple dynamic model for a two-component label decay in liver mitochondria**

Equations representing the model, describing fast (exponential, 6-^14^C arginine dependent) and slow (linear, non-specific ^14^C labels) degradation of ^14^C labelled mitochondria are given in the main paper.

An advantage to this method over the least squares method, which contains an implicit assumption of normally distributed error, is that we can specify any error model we wish. We have repeated this analysis with both normal and lognormal error models. The choice of error model in this case makes no difference to the conclusions, and we present the normal error model here for clarity.

In order to perform Bayesian inference, we must specify prior distributions on the parameters in our dynamic model and our error model. These were our best guesses of distributions for these parameters based on examining the data by eye, and constrained by our biological knowledge about the system:

$$\begin{matrix} y_{j}^{0}\sim N(500.0,0.0001) \\ k_{j}^{d}\sim N(0.5,100) \\ m_{j}^{k}\sim N(-2.0,1.0) \\ c_{j}^{k}\sim N(75.0,0.005)\text{ }\forall j=DR,Control \\ \tau\sim\Gamma(0.025,0.5)\text{ }\forall k=Brain,Muscle \end{matrix}$$

Where *N(a,b)* signifies a normally distributed parameter with expected value *a* and variance *1/b*. Note that in OpenBUGS the probability density function for the gamma distribution (chosen here since it always returns a positive value) is defined as follows:

$$f(x;r,\theta)=\frac{\theta^{r}x^{r-1}e^{-\theta x}}{\Gamma(r)}$$

$$\begin{matrix} Brain_{j}^{i}\sim N(\mu_{_{j}}^{Brain}(t^{i}),\tau)\text{ } \\ Muscle_{j}^{i}\sim N(\mu_{_{j}}^{Muscle}(t^{i}),\tau)\text{ } \\ Liver_{j}^{i}\sim N(\mu_{_{j}}^{Liver}(t^{i}),\tau)\text{ }\forall j=DR,Control \end{matrix}$$

Where *Brain_j_^i^*, *Muscle_j_^i^* and *Liver_j_^i^* are the measurements observed at time point *i* in the brain, muscle and liver in population *j* respectively. τ is a shape parameter for the modelled error distribution.

We assume that the precision of the error distribution for all measurements is the same.

In order to facilitate direct comparison with half-lives (λ) found in the literature, we can calculate label half-lives analytically for this model for each simulation as follows:

$$\lambda=\frac{-ck^{d}+k^{d}y^{0}+2m*W\left(\frac{-e^{\frac{k^{d}(c-y^{0})}{2m}}k^{d}y^{0}}{m}\right)}{2k^{d}m}$$

The function W calculates the principal value of the Lambert-W function. It was calculated in this analysis using the ProductLog function in Mathematica 5.0 (Wolfram Research Inc, 2003).

The OpenBugs script (which includes the raw experimental data) used for parameter inference for this model can be found here:

www.cisban.ac.uk/downloads/Miwa2008.odc

**References**

Cadenas S, Echtay KS, Harper JA, Jekabsons MB, Buckingham JA, Grau E, Abuin A, Chapman H, Clapham JC, Brand MD (2002) The basal proton conductance of skeletal muscle mitochondria from transgenic mice overexpressing or lacking uncoupling protein-3. *J. Biol. Chem.* **277**, 2773-2778.

Chappell JB, Hansford RG (1972) Preparation of mitochondria from animal tissues and yeasts. In *Subcellular components: Preparation and fractionation* (Bernie GD, ed) London: Butterworth, pp. 77-91.

Lai JC, Clark JB (1979) Preparation of synaptic and nonsynaptic mitochondria from mammalian brain. *Methods Enzymol.* **55**, 51-60.

McFarlane AS (1963) Measurement of Synthesis Rates of Liver-Produced Plasma Proteins. *Biochem. J*. **89**, 277-290.

Millward DJ (1970) Protein turnover in skeletal muscle. I. The measurement of rates of synthesis and catabolism of skeletal muscle protein using ^14^C Na_2_CO_3_ to label protein. *Clin. Sci*. **39**, 577-590.

Swick RW (1958) Measurement of protein turnover in rat liver. *J. Biol. Chem*. **231**, 751-764.

Swick RW, Ip MM (1974) Measurement of protein turnover in rat liver with ^14^C carbonate. Protein turnover during liver regeneration. *J. Biol. Chem.* **249**, 6836-6841.

Swick RW, Song H (1974) Turnover rates of various muscle proteins. *J. Anim. Sci.* **38**, 1150-1157.

Wolfram Research Inc (2003) *Mathematica*, Champaign, IL.
