# Supplementary material for: Mitochondrial turnover in liver is fast in vivo and is accelerated by dietary restriction: application of a simple dynamic model
Source: Aging Cell. 2008 Dec;7(6):920–3. doi: 10.1111/j.1474-9726.2008.00426.x (PMC2659384; doi:10.1111/j.1474-9726.2008.00426.x)
Supplement: Supplementary file 2 [file ace0007-0920-SD2.doc]

**Supplementary Table 1**. **Parameter estimates and their distribution statistics.**

| Parameter | Mean | Standard Deviation | 2.5% Quantile | Median | 97.5% Quantile | Frequency (%)  DR > Control |
| --- | --- | --- | --- | --- | --- | --- |
| $c_{Control}^{Brain}$(count.mg^-1^) | 57.9 | 6.8 | 44.3 | 57.9 | 71.2 |  |
| $c_{DR}^{Brain}$(count.mg^-1^) | 106.2 | 7.0 | 92.6 | 106.2 | 119.8 | 0* |
| $c_{Control}^{Muscle}$(count.mg^-1^) | 51.9 | 7.6 | 36.8 | 51.9 | 67.3 |  |
| $c_{DR}^{Muscle}$(count.mg^-1^) | 64.8 | 7.2 | 50.6 | 64.8 | 78.8 | 89.35 |
| $c_{Control}$(count.mg^-1^) | 54.9 | 5.3 | 44.3 | 54.8 | 65.5 |  |
| $c_{DR}$(count.mg^-1^) | 85.5 | 5.0 | 75.7 | 85.5 | 95.4 | 100* |
| $k_{Control}^{d}$ (d^-1^) | 0.4 | 0.0 | 0.3 | 0.4 | 0.5 |  |
| $k_{DR}^{d}$ (d^-1^) | 0.6 | 0.0 | 0.5 | 0.6 | 0.7 | 99.96* |
| $m_{Control}^{Brain}$ (count.mg^-1^.d^-1^) | -2.4 | 0.8 | -3.8 | -2.4 | -0.9 |  |
| $m_{DR}^{Brain}$ (count.mg^-1^.d^-1^) | -1.9 | 0.7 | -3.3 | -1.9 | -0.4 | 68.43 |
| $m_{Control}^{Muscle}$ (count.mg^-1^.d^-1^) | -2.4 | 0.8 | -3.9 | -2.4 | -0.8 |  |
| $m_{DR}^{Muscle}$ (count.mg^-1^.d^-1^) | -2.0 | 0.7 | -3.5 | -2.0 | -0.6 | 62.04 |
| $m_{Control}$ (count.mg^-1^.d^-1^) | -2.4 | 0.5 | -3.4 | -2.4 | -1.3 |  |
| $m_{DR}$ (count.mg^-1^.d^-1^) | -1.9 | 0.5 | -2.9 | -2.0 | -1.0 | 71.91 |
| $\mu_{Control}^{0}$ (count.mg^-1^) | 313.3 | 19.7 | 277.9 | 312.7 | 354.2 |  |
| $\mu_{DR}^{0}$ (count.mg^-1^) | 651.6 | 45.6 | 563.5 | 651.4 | 742.2 | 100* |
| $\lambda_{Control}$ (d) | 2.2 | 0.2 | 1.8 | 2.2 | 2.7 |  |
| $\lambda_{DR}$ (d) | 1.4 | 0.1 | 1.2 | 1.4 | 1.6 | 0.03* |
| $\lambda_{Control}^{Fast}$ (d) | 1.8 | 0.2 | 1.5 | 1.8 | 2.2 |  |
| $\lambda_{DR}^{Fast}$ (d) | 1.2 | 0.1 | 1.0 | 1.2 | 1.3 | 0.035* |
| $\tau$ | 0.0033 | 0.0007 | 0.0022 | 0.0033 | 0.0048 |  |

Statistical summaries of posterior model parameter distributions from 20,000 updates of the MCMC method implemented in OpenBUGS (Thomas et al. 2006). Convergence of the method was ensured by first performing 10,000 updates and discarding these simulations as “burn-in”. The frequency column summarises the percentage of the 20,000 converged OpenBUGS simulations in which the parameter value relevant to DR is greater than that relevant to the control experiment. Asterisks signify differences significant at the 95% level (>95% if the DR value is greater than the control value, <5% if the converse is true).
